# Enhancement of luminescence in white emitting strontium fluoride core @ calcium fluoride shell nanoparticles

**DOI:** 10.1186/s11671-015-1053-6

**Published:** 2015-09-01

**Authors:** Nandini Kumam, Ningthoujam Premananda Singh, Laishram Priyobarta Singh, Sri Krishna Srivastava

**Affiliations:** Department of Chemistry, Manipur University, Imphal, 795003 India

**Keywords:** Core shell fluoride nanomaterials, Luminescence, White emitter

## Abstract

Synthesis of lanthanide-doped fluoride SrF_2_:3Dy and SrF_2_:3Dy@CaF_2_ nanoparticles with different ratios of core to shell (1:0.5, 1:1 and 1:2) has been carried out by employing ethylene glycol route. X-ray diffraction (XRD) patterns reveal that the structure of the prepared nanoparticles was of cubical shape, which is also evident in TEM images. The size of the nanoparticles for core (SrF_2_:3Dy) is found to increase when core is covered by shell (CaF_2_). It is also evident from Fourier transform infrared spectroscopy (FTIR) that ethylene glycol successfully controls the growth and acts as a shape modifier by regulating growth rate. In the photoluminescence investigation, emission spectra of SrF_2_:3Dy is found to be highly enhanced when SrF_2_:3Dy is covered by CaF_2_ due to the decrease of cross relaxation amongst the Dy^3+^-Dy^3+^ ions. Such type of enhancement of luminescence in homonanostructure SrF_2_:3Dy@CaF_2_ (core@shell) has not been studied so far, to the best of the authors’ knowledge. This luminescent material exhibits prominently white light emitting properties as shown by the Commission Internationale d’Eclairage (CIE) chromaticity diagram. The calculated correlate colour temperature (CCT) values for SrF_2_:3Dy, SrF_2_:3Dy@CaF_2_ (1:0.05), SrF_2_:3Dy@CaF_2_ (1:1) and SrF_2_:3Dy@CaF_2_ (1:2) are 5475, 5476, 5384 and 5525 K, respectively, which lie in the cold white region.

**Graphical abstract:**
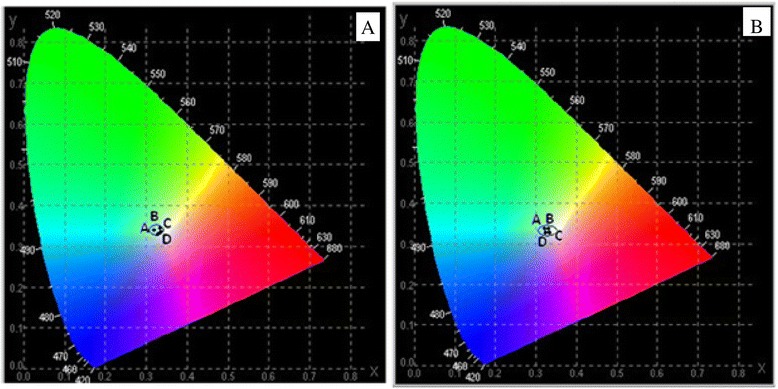
White light emitting homonanostructure material SrF_2_:3Dy@CaF_2_(core@shell).

White light emitting homonanostructure material SrF_2_:3Dy@CaF_2_(core@shell).

## Background

Fluorides have wide applications in optics as windows, lenses and scintillation crystals and also exhibit interesting properties in optoelectronics such as lasing [1, 2], light amplification, and upconversion [3–5] as host crystals for lanthanide ions. The solid fluoride materials have drawn attention due to their unique properties, like low-energy phonons, high ionicity, electron-acceptor behaviour, high resistivity and anionic conductivity [6–8] and also have wide range of potential optical applications in optics, biological labels and lenses [9, 10]. Fluoride base system possesses very low vibrational energy comparing oxide-base system therefore quenching of excite states of the lanthanide ions is minimal in fluoride-based system [11, 12].

Strontium fluoride (SrF_2_) has immense applications in microelectric and optoelectric devices because of its dielectric properties and is used as an attractive host for phosphors [13, 14]. There are two main fluorescence transitions of trivalent dysprosium (Dy^+3^ ion) having 4f^9^ electronic configuration, which are ^4^F_9/2_ → ^6^H_15/2_ (blue) and ^4^F_9/2_ → ^6^H_13/2_ (yellow-orange wavelength region) [15, 16]. ^4^F_9/2_ → ^6^H_13/2_ is hypersensitive transition and strongly depend on crystal-field environment. White light emission can also achieved by doping with lanthanide ions (Ln^3+^) which emit primary colour (red, blue and green). White emitter characteristics are also exhibited by nanomaterials prepared by surfactant route with multiple lanthanide doping [17]. However, Dy^3+^ ion emits white light at a suitable yellow-to-blue intensity ratio [18, 19].

Enhancement of luminescence intensity of the lanthanide-doped nanoparticles has potential applications in optoelectronic devices. A core-shell formation in lanthanide-doped nanoparticles is one of the useful methods for enhancing luminescence.

In this present work, we have reported dysprosium ion (3 at.% )-doped SrF_2_ (SrF_2_:3Dy) nanoparticles and studied its photoluminescence properties. In order to enhance luminescence intensity, SrF_2_:3Dy (core) is covered by CaF_2_ (shell) at different ratios of shells (1:0.5, 1:1, 1:2). The Commission Internationale d’Eclairage (CIE) colour coordinates are found to be very close to ideal white values.

## Methods

### Chemicals and reagents

Strontium nitrate (Sr(NO_3_)_2_, 99.9 %), dysprosium (III) nitrate pentahydrade (Dy(NO_3_)_3_·5H_2_O, 99.99 %), from Sigma-Aldrich, ethylene glycol, ammonium fluoride NH_4_F and calcium chloride, CaCl_2_ from Merck, and methanol were used as received without further purification. Double distilled water was used throughout the experiment.

### Preparation of SrF_2_:3Dy and SrF_2_:3Dy core @CaF_2_ shell nanoparticles

Pure SrF_2_ and Dy^3+^ ion (3 at.%)-doped SrF_2_ nanoparticles were synthesised by ethylene glycol route. In a typical synthesis procedure, stoichiometric amount of precursor of Dy^3+^ ions and strontium nitrate were heated at 40 °C for 30 min and ammonium fluoride was added. The resulting precipitate solution was allowed to mix with 50 ml of ethylene glycol and refluxed at 120 °C for 2 h. The resulting white precipitate was collected by centrifugation at 10,000 rpm after washing with methanol. In case of core-shell, i.e., SrF_2_:3Dy@CaF_2_ (1:0.5), SrF_2_:3Dy@CaF_2_ (1:1) and SrF_2_:3Dy@CaF_2_ (1:2), once the above procedure of lanthanide doping is done, requisite amount of calcium chloride solution is added and refluxed at 40 °C for about 30 min. Further, ammonium fluoride solution is added, and whole content is refluxed again at 120 °C about 2 h.

### Characterisation of nanoparticles

Philips x-ray diffractometer (PW 1071) with Cu*k*_*α*_ (1.5 Å) radiation having Ni filter was used for x-ray diffraction (XRD) study. All patterns were recorded over the angular range 10 ≤ 2*θ*/deg ≤ 70 with a step size of ∆2*θ* = 0.02. The powder samples were ground and dispersed in methanol on a glass slide and allowed to dry. The average crystallite size and strain were calculated using Williamson-Hall model. The lattice parameters were calculated from the least square fitting of the diffraction peaks.

For the characterisation of the nanoparticles, Fourier transform infrared spectroscopy (FTIR) spectra were recorded on a Perkin Elmer Spectrum 400 FT-IR spectrometer. Powder samples were studied by making thin pellets with KBr.

JEOL 2000 FX transmission electron microscope was used for recording TEM images. For the TEM measurement, the powder samples were ground and dispersed in methanol. A drop of the dispersed particles was put over the carbon coated copper grid and evaporated to dryness at room temperature.

All the luminescence spectra were recorded using LS-55 Photoluminescence Spectrometer. Powder samples were dispersed in methanol and spread over the quartz slide and dried at room temperature.

## Results and discussion

### X-ray analysis

**Fig. 1:**
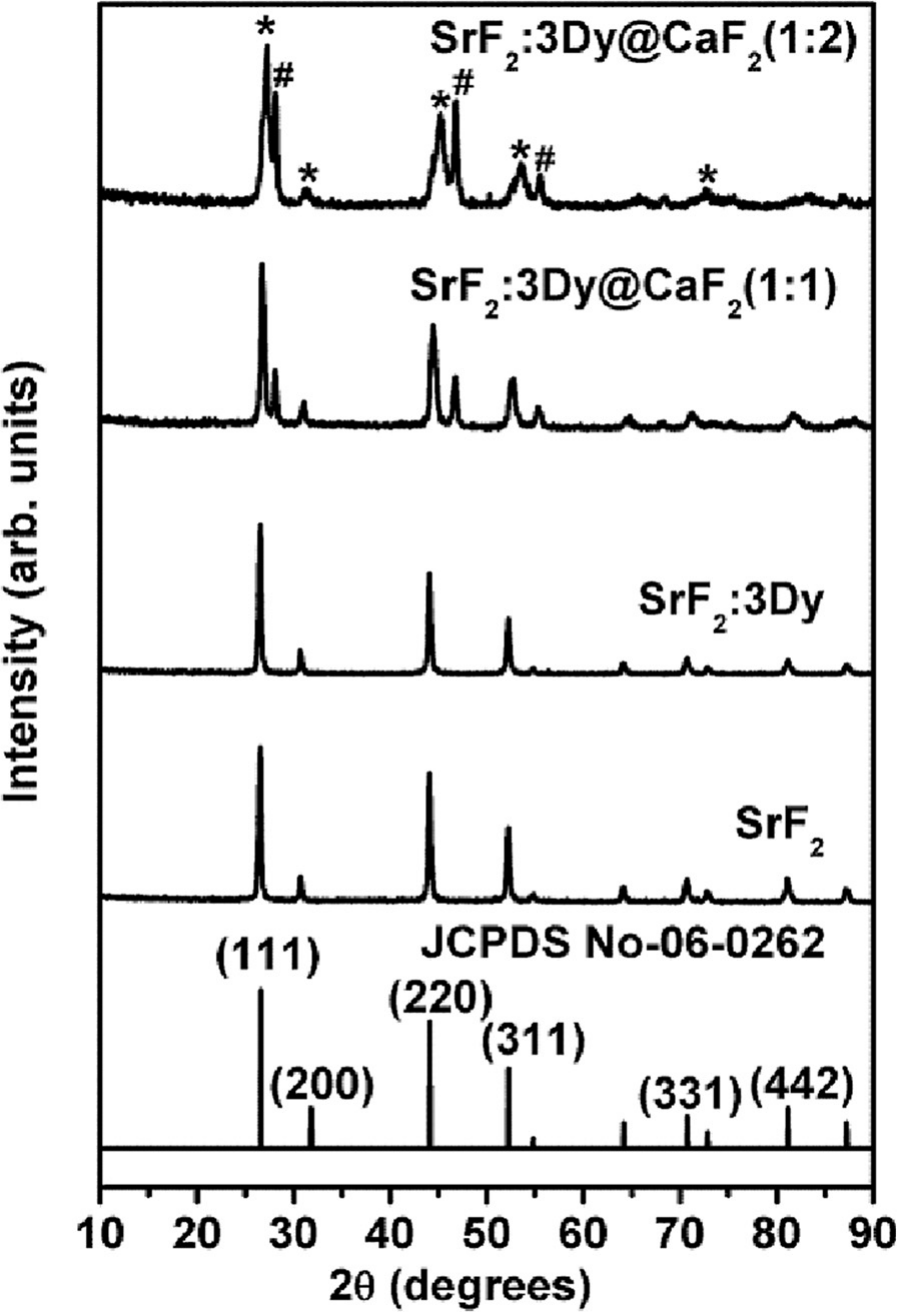
XRD pattern of pure, doped (3 at.% Dy^3+^ ion) SrF_2_ core and CaF_2_ shell at different ratios of shell

Figure 1 shows the XRD pattern of SrF_2_, SrF_2_:3Dy and SrF_2_:3Dy@CaF_2_ at two different ratios of shell (1:1 and 1:2). All the peaks pattern for pure and SrF_2_:3Dy can be index with the Joint Committee on Powder Diffraction Standards (JCPDS) card number 06-0262 having cubical structure with space group Fm3m (225). In case of SrF_2_:3Dy@CaF_2_ core-shell, the peak patterns are found coexisting for both SrF_2_ and CaF_2_ phases, indicating the CaF_2_ coated on the SrF_2_.The star mark in the Fig. 1 indicates the SrF_2_ phase,and the hutch mark indicates the CaF_2_ phases. For CaF_2_, the peaks patterns are found to be face centre cubical structure with space group Fm3m (225) (JCPDS No. 35-0816). All the prepared samples are found to be crystalline structure, and there is no extra impurity peaks from the precursors of core SrF_2_ and shell CaF_2_. The lattice parameter and unit cell volume of the prepared samples are given in the Table 1. Williamson-Hall model [20] has been used to calculate the microstrain (ε) for the system under study.

**Table 1:** Lattice parameter, unit cell volume and crystallite sizes of SrF_2_, SrF_2_:3Dy and SrF_2_:3Dy@CaF_2_ nanoparticles

Sl.	Sample	Unit cell (Å)	Volume (Å^3^)
1	JCPDS No.06-0262	5.80	195.11
2	SrF_2_	5.79	194.96
3	SrF_2_:3Dy	5.8062	195.7365
4	SrF_2_:3Dy @CaF_2_(1:1)	5.7899	194.0946
5	SrF_2_:3Dy@CaF_2_(1:2)	5.7374	188.8651

1 $$ \beta \cos \uptheta = 0.9\lambda /D + 4\upvarepsilon\  \sin \uptheta $$

Here, *β* is the full width at half maxima (FWHM) at Bragg’s angle (2*θ*), with *λ* being the x-ray wavelength (CuK_α_ = 1.54 Å). The calculated crystallite sizes are in the ranges of 20–40 nm (Table 2). From the Table 1, we observed that the unit cell volumes of the SrF_2_:3Dy is larger than SrF_2_ this can be explained as follows. The ionic radii of the Sr^2+^ (r_Sr_^2+^ = 1.27 Å) [21] is larger than the size of Dy^3+^ ( r_Dy_^3+^ = 1.03 Å) [22], and according to Vegard’s law, incorporation of Dy^3+^ ion in SrF_2_ results in shrinking of unit cell volume as compared to the standard data (JCPDS No. 01-1274). But from the data (lattice parameter, unit cell volume and crystallite sizes of SrF_2_, SrF_2_:3Dy and SrF_2_:3Dy@CaF_2_ nanoparticles) given in Table 1, we observed that there is slight increase in unit cell volume from the reported value. This is due to the formation of the interstitials fluoride ions, i.e., when Sr^2+^ is substituted by a trivalent Dy^3+^ ion, excess positive charges are compensated by interstitial fluoride ions where electronic repulsion between F^−^ ions also exist leading to the increase in unit cell volume [23] which indicates the incorporation of the Dy^3+^ ion into the SrF_2_ crystal lattice. Full width at half of maximum values for SrF_2_, SrF_2_:3Dy and SrF_2_:3Dy@CaF_2_ (1:1 and 1:2) are 0.24696, 0.26582, 0.3437 and 0.52442° in 2*θ*. The full width at half-maximum values increased gradually, which indicated the decrease of crystallinity (decreasing of crystallite size) and increase of disorder extent. Decrease in crystallinity of the SrF_2_:3Dy when covered with CaF_2_ can explained as there is possibility of substitution of some Sr^2+^ ions with Ca^2+^ ions (r_Ca_^2+^ = 1.50 Å) because they are almost having similar sizes and same charges. This type of substitution creates deformation in the crystal, which results in decreases of peak sharpness. Further, when SrF_2_:3Dy is covered with CaF_2_, there is decrease in penetration depth of x-ray passing through core (SrF_2_). This is reflected in the intensity of peaks corresponding to SrF_2_ of core-shell to decrease when compared to SrF_2_ without covering shell. The unit cell volume also decreases when SrF_2_:3Dy is covered with CaF_2_ which can be explained as, some of Dy^3+^ ion located near the surface gets distributed into CaF_2_ leading to decrease in electronic repulsion between F^—^ ions and unit cell volume decreases. Moreover, with increased coating of CaF_2_ over SrF_2_ particle, there is decrease of penetration depth of x-ray passing through the core. This results in decrease of intensity of peaks corresponding to SrF_2_, and this is possible only when SrF_2_ is cover by CaF_2_ rather than they are existing as two separated phases.

**Table 2:** The strain and crystallite size for of SrF_2_, SrF_2_:3Dy and SrF_2_:3Dy@CaF_2_ nanoparticles

Sl no.	Samples	Strain	Size (nm)
1	SrF_2_	0.00397	30
2	SrF_2_:3Dy	0.00432	37
3	SrF_2_:3Dy@CaF_2_ (1:0.5)	3.6888E-4	32
4	SrF_2_:3Dy@CaF_2_ (1:1)	2.6888E-4	31
5	SrF_2_:3Dy@CaF_2_ (1:2)	2.4534E-4	25

### FTIR study

**Fig. 2:**
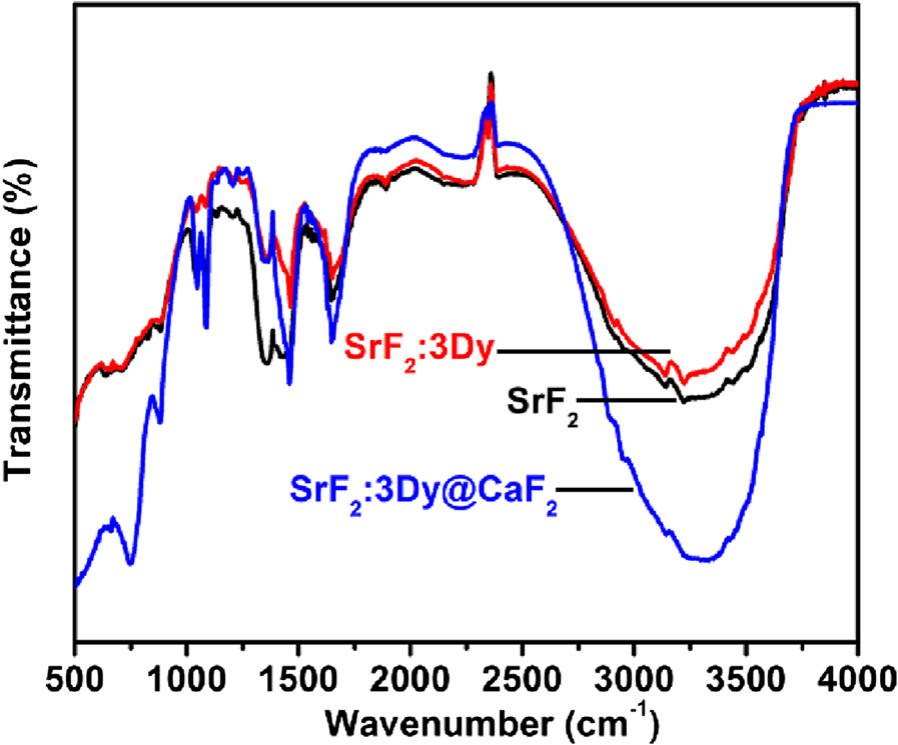
FTIR spectrum of SrF_2_, SrF_2_:3Dy and SrF_2_:3Dy@CaF_2_ nanoparticles

Figure 2 shows the FTIR spectra of SrF_2_, SrF_2_:3Dy core and its shell with CaF_2_ nanoparticles at different ratios of shell component. FTIR spectra of ethylene glycol is attribute by O–H stretching and bending vibrations around 3300–3600 cm^−1^ and 1500–1600 cm^−1^; the broadening around 3300–3600 cm^−1^ is due to the O-H stretching vibration. Stretching vibration corresponds to C–O and C–C linkage appearing around 1000–1100 cm^−1^ and 1000–1200 cm^−1^, respectively. The C–C vibration is dominant over the C–O vibration. The peak around 2900 cm^−1^ has been corresponds to the C–H stretching vibrations [24]. The FTIR pattern of SrF_2_ nanoparticles shows the peak characterise of O–H, C–C, C–O and C–H indicating that the nanoparticles are capped by ethylene glycol molecule which was used as capping agent.

### TEM study

**Fig. 3:**
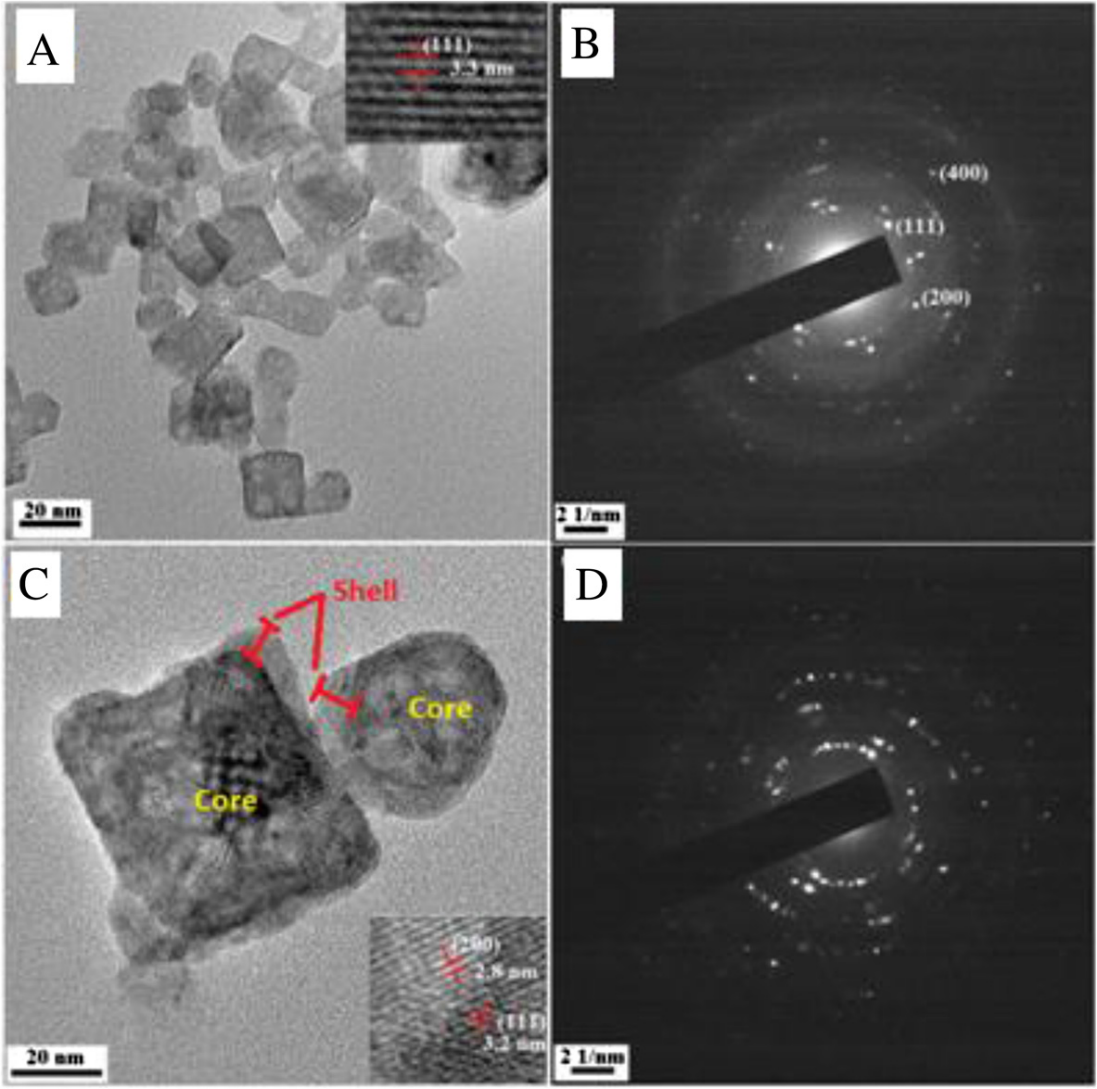
**a** TEM image of SrF_2_ :3Dy (core) and **b** its SAED pattern. **c** TEM image of SrF_2_:3Dy@CaF_2_ (1:2) and **d** its SAED pattern

TEM image of SrF_2_:3Dy (core) shows that the prepared nanoparticles are of cubical shape and some spherical particles (Fig. 3a), and the core particles size are in the range of 8–30 nm. HRTEM image (upper inset Fig. 3a) gives the value of *d* = 3.3 Å which is correspond to (111) plane of SrF_2_:3Dy according to JCPDS No. 06-0262. The selected area electron diffraction (SAED) pattern of SrF_2_:3Dy (Fig. 3b) shows crystallinity of the prepared nanoparticles exhibiting clear spots on the rings. In case of SrF_2_:3Dy@CaF_2_ (core@shell) with the ratio 1:2, the shape of the particles is still cubical and spherical (Fig. 3c). The size of the core@shell nanoparticles were found to be larger than core where the size of the cubical shaped core@shell particles is 50 nm and that of spherical is 37 nm. The cubical shape appears to be covered by another cube, but image is not clear and similar thing happens in case of spherical particles also. This indicates that CaF_2_ shell covers the SrF_2_:3Dy core. From the HRTEM image (lower inset Fig. 3c), we observed two kinds of plane, one gives the value of *d* = 2.8 Å which corresponds to (200) plane of core (SrF_2_:3Dy) according to JCPDS No. 06-0262, and the other one gives the value of *d* = 3.2 Å corresponding to (111) plane of shell (CaF_2_) according to JCPDS No. 35-0816. These data indicated that core (SrF_2_:3Dy) is well covered by shell (CaF_2_). The SAED pattern of core@shell is also shown in Fig. 3d, and particles are still crystalline.

**Fig. 4:**
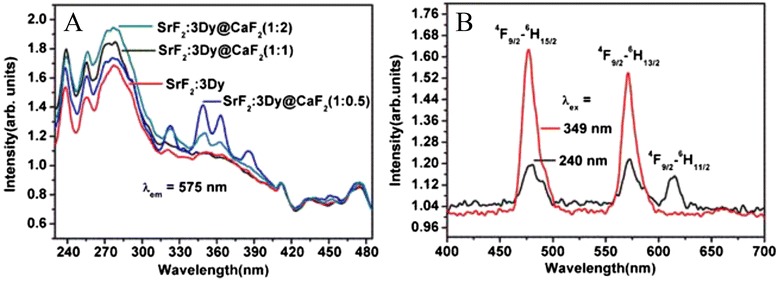
**a** TEM image of shell CaF_2_ (inset HRTEM of shell) and **b** SAED of shell

In order to see the crystallinity of shell CaF_2_, the TEM image and SAED pattern of shell alone are shown in Fig. 4. The shape of shell is found to be cubic (Fig. 4a) with particle size of 20 nm. The HRTEM of shell (inset Fig. 4a) clearly indicates crystallinity of CaF_2_ and gives the value of *d* = 1.9 Å which corresponds to (220) plane of CaF_2_ according to JCPDS No. 35-016. In the SAED pattern also (Fig. 4b), the spots in the rings indicate crystallinity of shell and diffraction rings are labelled accordingly.

### Photoluminescence study

**Fig. 5:**
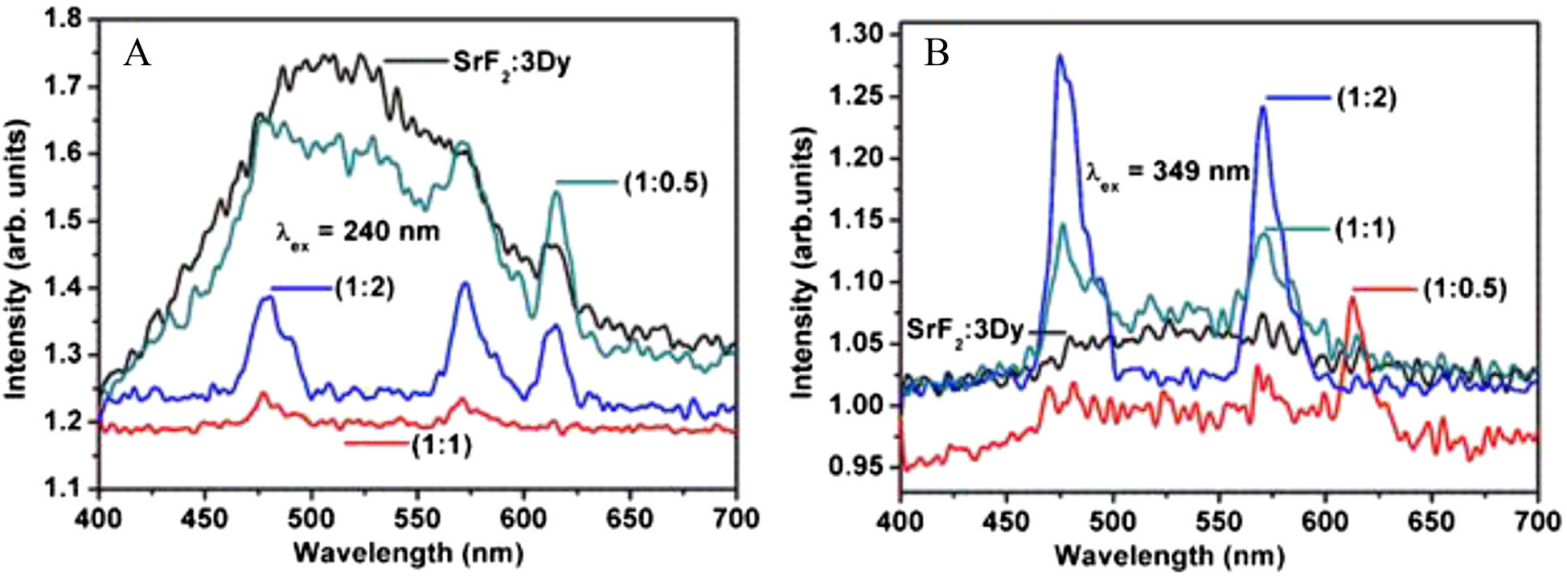
**a** Excitation spectra of SrF_2_:3Dy core nanoparticles and its CaF_2_ shell at different ratios of shells monitor at 575-nm emission wavelength. **b** Emission spectra of SrF_2_:3Dy@CaF_2_ (1:2) monitor at two different excitation wavelengths 240 and 349 nm

Figure 5a shows the excitation spectra of the SrF_2_:3Dy and SrF_2_:3Dy@CaF_2_ at two different ratios of shell monitored at 575-nm emission wavelength. In order to study the excitation spectra, we can divide the whole spectrum into two regions, one in the range 200–280 nm and another is 280–400 nm. The region from 200–280 nm corresponds to the transition from 2p orbital of F^−^ ions to the 4f orbital of Dy^3+^ ions (charge transfer transition) and consists of peaks at 239, 256 and 277 nm. The peaks from 280–400 nm are related with the 4f-4f transition of Dy^3+^ ion. The peak at 349, 363, 386, 435, 453 and 474 nm can be assigned to the transition from ^6^H_15/2_ to ^6^P_7/2 ,_^6^P_5/2_ , ^4^I_13/2_ , ^4^G_11/2 ,_^4^I_15/2_ and from ^4^F_9/2_ to ^4^H_15/2_ of Dy^3+^ ion [25].

The emission spectra monitored at two different excitation wavelengths 240 and 349 nm (Fig. 5b) for the SrF_2_:3Dy@CaF_2_ (1:2) nanoparticles shows the characteristic emission peaks of Dy^3+^ ion at 475 and 570; 612 nm originating from ^4^F_9/2_ → ^6^H_15/2_ (blue) and ^4^F_9/2_ → ^6^H_13/2_ (yellow-orange), as we know that the yellow part of Dy^3+^ ion is in the range extended from 560–610 nm [22]. So 612-nm peak is related to the yellow part of the Dy^3+^ ion and consists of some red part also. The peak of 612-nm emission is seen in case of 240-nm excitation, but for 349-nm excitation, only two peaks at 475 and 570 nm are seen since the intensity of the peaks are higher when excited at host (charge transfer transition), indicating good energy transfer from host to Dy^3+^ ion. In case of 349 nm excitation, the peak of 570 nm emission is predominant so the peak of 612 nm cannott be seen. The peak at 475 nm is most intense as compared to other peaks.

**Fig. 6:**
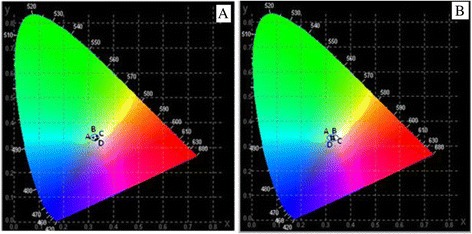
Emission spectra of SrF_2_:3Dy core nanoparticles and its CaF_2_ shell at different ratios of shells excited at **a** 240 nm and **b** 349 nm

In case of all nanoparticles excited at 240 nm (Fig. 6a), the peaks at 475 nm, i.e. ^4^F_9/2_ → ^6^H_15/2_ transition, are most intense and they have same peak pattern except their intensities are different. From the figure, we observed that the intensity of the peak for the core SrF_2_:3Dy is less than the intensity of the peak for core@shell SrF_2_:3Dy@CaF_2_ and increase in intensity is also seen for the emission peaks of Dy^3+^ ion with increasing concentration of the shell. The intensity of the emission peaks increases when core (SrF_2_:3Dy) is covered with CaF_2_ (shell); this can be explained as, Dy^3+^ ion have strong cross relaxation amongst Dy^3+^-Dy^3+^ ions resulting in decrease in luminescence intensity. But SrF_2_:3Dy is covered by CaF_2_ where some part of Dy^3+^ ion is diffused to the CaF_2_ so that Dy^3+^ ion concentration decreases resulting in decreases of the cross relaxation amongst the Dy^3+^-Dy^3+^ ions leading to increase in emission intensity. Moreover –OH present on the surface of the core is less in number when core is covered by shell. Because of the removal of –OH/dangling bond from the surface of the core, the non-radiative decay diminishes, and hence, the emission intensity increases when core is covered by shell. We know that –OH decreases luminescence intensity through multiphonon relaxation [17]. Enhancement of emission intensity can also be assigned to decreasing tensile strain value as reported elsewhere [26], and this has been also observed in our prepared nanoparticles. Tensile strain value for core@shell is decreased as compared to core itself. The values of strain are given in Table 2. Because of above factors, the emission intensity of SrF_2_:3Dy (core) is enhanced when it is covered by shell (CaF_2_).

Upon excitation at 240 nm, the emission spectrum of core SrF_2_:3Dy is broadened. This is related to defect-induced emission from host. There is large difference in ionic sizes in Sr^2+^ and Dy^3+^, which creates defects in lattice. When shell of CaF_2_ is introduced, some parts of Dy^3+^ occupy Ca^2+^ sites. Ionic size difference between Ca^2+^ and Dy^3+^ is much less than that between Sr^2+^ and Dy^3+^. Thus, defect in lattice decreases after formation of core@shell. The emission peak intensity arising from defects decreases. In case of core@shell (1:2), broad emission peak almost disappears; instead, emission peaks of Dy^3+^ are observed. Emission spectrum gives sharp peaks corresponding to Dy^3+^ when excitation is chosen at 349 nm (direct excitation) irrespective of samples (core or core@shell) as shown in Fig. 6b. It means that 349-nm excitation cannot create electron-hole pairs from lattice defects. The energy associated with 349 nm is much less than that of 240 nm, and thus, it is insufficient to generate electron-hole pairs. In case of 240-nm CT excitation, there are various ways of excited electrons returning to the ground state, and in the meantime, energy diffusion may also occur. But in the case of 349-nm direct excitation, there is a limited path for returning of excited electron to ground state [27]. The emission intensity of the 240-nm excitation is slightly less than that of 349-nm excitation indicating that energy transfer from charge transfer band is less than direct excitation. Such type of enhancement of luminescence in homonanostructure SrF_2_:3Dy@CaF_2_ (core@shell) has not been studied so far, to the best of the authors’ knowledge.

**Fig. 7:**
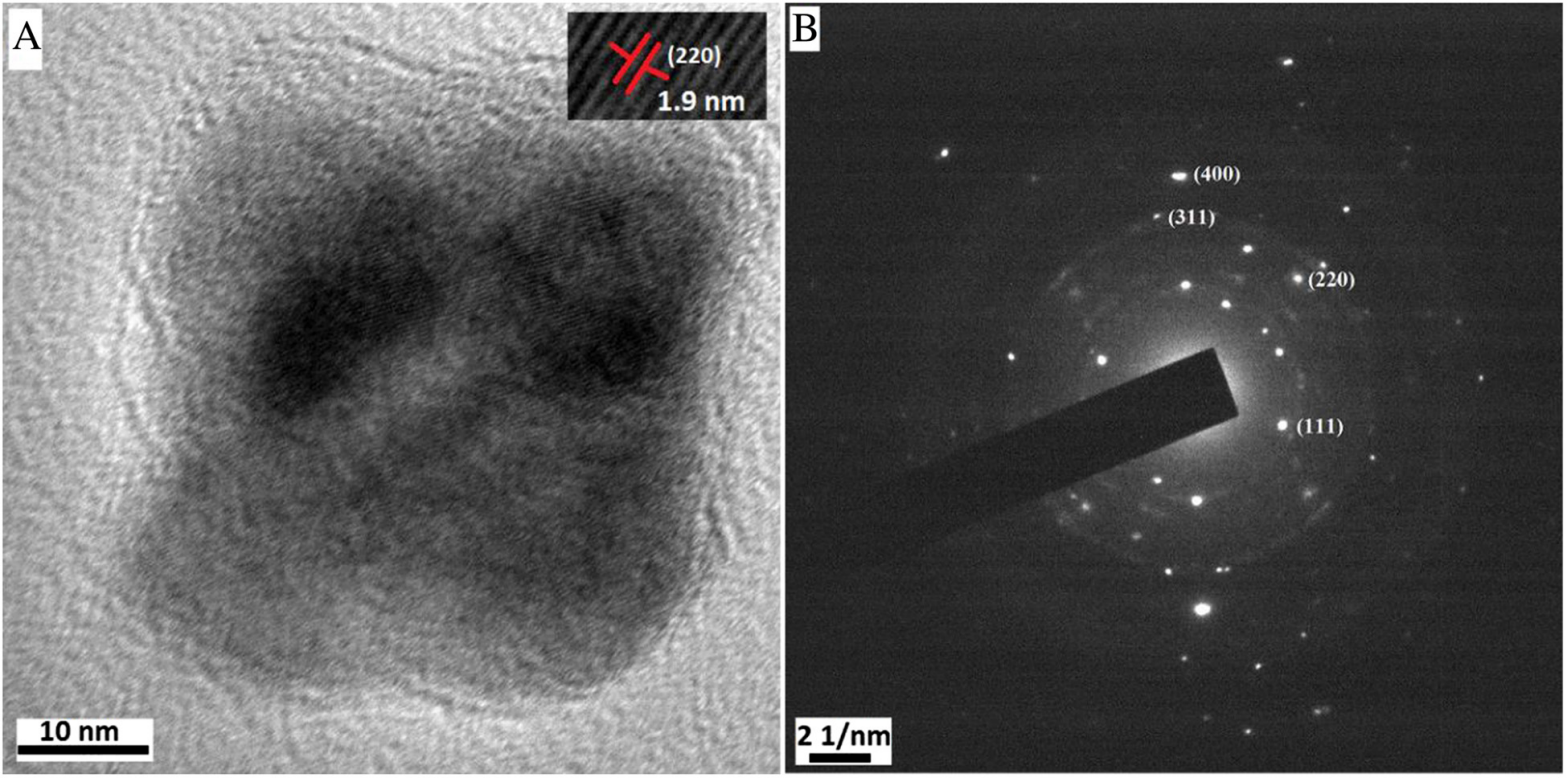
CIE diagram of SrF_2_:3Dy, SrF_2_:3Dy@CaF_2_ (1:0.5, 1:1 and 1:2) nanoparticles excited at two different excitation wavelengths **a** 240 and **b** 349 nm

Figure 7 shows the Commission Internationale d’Eclairage (CIE) coordinate diagram of SrF_2_:3Dy and SrF_2_:3Dy@CaF_2_ at different ratios excited at two different excitation wavelengths 240 and 349 nm emitting white light. The corresponding chromaticity coordinates for 240-nm excitation (Fig. 7a) are (A) SrF_2_:3Dy (*x* = 0.334, *y* = 0.348), (B) SrF_2_:3Dy@CaF_2_ (1:0.5) (*x* = 0.338, *y* = 0.346), (C) SrF_2_:3Dy@CaF_2_ (1:1) (*x* = 0.333, *y* = 0.334) and (D) SrF_2_:3Dy@CaF_2_ (1:2) (*x* = 0.334, *y* = 0.335) and for 349-nm excitation (Fig. 7b) are (A) SrF_2_:3Dy (*x* = 0.333, *y* = 0.337), (B) SrF_2_:3Dy@CaF_2_ (1:0.5) (*x* = 0.333, *y* = 0.338), (C) SrF_2_:3Dy@CaF_2_ (1:1) (*x* = 0.335, *y* = 0.336) and (D) SrF_2_:3Dy@CaF_2_ (1:2) (*x* = 0.331, *y* = 0.334). The calculated chromaticity coordinates are very close to the other reported values [28]. The quality of the white light calculated by using Mc Camy empirical formula [29] in terms of correlate colour temperature (CCT) values, which is given below:

$$ \mathrm{C}\mathrm{C}\mathrm{T} = -449{n}^3 + 352\ {n}^2\hbox{--}\ 6823n+5520.33 $$

Where, $n = \frac{x - {x}_c}{y - {y}_c}$ is negative inverse slope line, *x*_c_ = 0.332, and *y*_c_ = 0.186. The CCT values for excitation at 240 nm, for SrF_2_:3Dy, SrF_2_:3Dy@CaF_2_ (1:0.05), SrF_2_:3Dy@CaF_2_ (1:1) and SrF_2_:3Dy@CaF_2_ (1:2) are 5436, 5264, 5474 and 5428 K, respectively. For excitation at 349 nm, the CCT values for SrF_2_:3Dy, SrF_2_:3Dy@CaF_2_ (1:0.05), SrF_2_:3Dy@CaF_2_ (1:1) and SrF_2_:3Dy@CaF_2_ (1:2) are 5475, 5476, 5384 and 5525 K, respectively. These CCT values lie in the cold white region. Thus, the prepared nanomaterials exhibit the characteristics of white emitter.

## Conclusions

Lanthanide ion (Dy^3+^)-doped SrF_2_ core and SrF_2_:3Dy@CaF_2_ shell at different ratios of shell (CaF_2_) have been successfully synthesised by using ethylene glycol as capping agent. The XRD pattern indicates that the synthesised nanoparticles have mainly cubical structure SrF_2_ (JCPDS 06-0262) and substitution of Dy^3+^ ion into SrF_2_ sites. Unit cell volume of the SrF_2_:3Dy decreases when it is covered by CaF_2_, since some parts of Dy^3+^ ions are distributed into the shell (CaF_2_) thereby decreasing both the electronic repulsion between F^—^ ions and unit cell volume. FTIR studies show the capping of SrF_2_ by ethylene glycol which is used as capping agent. The emission spectrum of the SrF_2_:3Dy core is weak due to the strong cross relaxation amongst the Dy^3+^-Dy^3+^ ions, which has been overcome by covering a layer of CaF_2_ shell over SrF_2_:3Dy (i.e., core@shell formation). Thus, in such core@shell system, the emission intensity of Dy^3+^ ion is enhanced effectively. Luminescence properties of such type of homonanostructure SrF_2_:3Dy@CaF_2_ have not been investigated so far, to the best of the authors’ knowledge. The emission spectra lies in the white region in CIE diagram and CCT calculated using Mc Camy empirical formula shows the prepared nanoparticles are found to be lying in the cold white region.
